# Quantitative Assessment of Trout Fish Spoilage with a Single Nanowire Gas Sensor in a Thermal Gradient

**DOI:** 10.3390/nano11061604

**Published:** 2021-06-18

**Authors:** Matteo Tonezzer, Nguyen Xuan Thai, Flavia Gasperi, Nguyen Van Duy, Franco Biasioli

**Affiliations:** 1 Research and Innovation Centre, Department of Food Quality and Nutrition, Fondazione Edmund Mach, Via E. Mach 1, 38098 San Michele all’Adige, Italy; flavia.gasperi@fmach.it (F.G.); franco.biasioli@fmach.it (F.B.); 2 Center Agriculture Food Environment, University of Trento/Fondazione Edmund Mach, Via E. Mach 1, 38010 San Michele all’Adige, Italy; 3 IMEM-CNR, Sede di Trento—FBK, Via alla Cascata 56/C, Povo, 38123 Trento, Italy; 4 International Training Institute for Materials Science, Hanoi University of Science and Technology, Hanoi 100000, Vietnam; xuanthai.vmi@gmail.com

**Keywords:** metal oxide, gas sensor, resistive sensor, single nanowire, fish spoilage, food freshness

## Abstract

The response of a single tin oxide nanowire was collected at different temperatures to create a virtual array of sensors working as a nano-electronic nose. The single nanowire, acting as a chemiresistor, was first tested with pure ammonia and then used to determine the freshness status of trout fish (*Oncorhynchus mykiss*) in a rapid and non-invasive way. The gas sensor reacts to total volatile basic nitrogen, detecting the freshness status of the fish samples in less than 30 s. The sensor response at different temperatures correlates well with the total viable count (TVC), demonstrating that it is a good (albeit indirect) way of measuring the bacterial population in the sample. The nano-electronic nose is not only able to classify the samples according to their degree of freshness but also to quantitatively estimate the concentration of microorganisms present. The system was tested with samples stored at different temperatures and classified them perfectly (100%), estimating their log(TVC) with an error lower than 5%.

## 1. Introduction

Microbial growth is important in food as it reduces the shelf life and increases the risk of foodborne illness. Fresh food is even more susceptible to this problem as it deteriorates rapidly and this affects not only the food industry but also the health of consumers, with social and health costs [1,2]. Production chains and distribution networks have expanded and become more complicated and this has increased the time by which food reaches the consumer [3].

Fish is a health food that is increasingly consumed around the world, often fresh or thawed [4]. Fish and fish products are considered “health food products” as they contain a large number of high-grade proteins (including all vital amino acids). Rainbow trout is a sustainable fish labeled a “best choice” by the EPA and FDA for its healthiness and low mercury content. Its consumption is widespread also thanks to the fact that it is a fish that lives in both fresh and marine water [5]. Rainbow trout production has grown exponentially since the 1950s, as reported by FAO statistics [6].

The quality of fresh fish is therefore a major concern for both the industry and consumers [7]. Initially, groups of human experts used to assess the appearance, smell and texture of the fish [8,9] but this procedure was laborious and time-consuming and therefore sensors capable of doing this automatically and objectively are being studied. Different methods have been used to evaluate the degree of freshness of the fish [8,10]. It is important that the sensor is small (portable), cheap (to deploy many along the production and distribution chain or to integrate one into the packaging) and fast (to measure in real time).

After the death of the fish, the microorganisms on its surface multiply and gradually spread to various tissues [11]. The proliferation of microbes is a major cause of fish spoilage. In fact, the total viable count (TVC) is commonly used as a reference and definitive index [12]. During this process, microbes degrade trimethylamine N-oxide (TMAO) into trimethylamine (TMA) and ammonia [13]. At the same time, bacteria decompose urea and amino acids and produce NH_3_ (ammonia) [14]. For these reasons, gas sensors usually measure total volatile basic nitrogen (TVB-N) consisting of ammonia, TMA and dimethylamine (DMA), which is commonly used as a freshness criterion for fish [15].

The most precise and accurate method to analyze volatile compounds is to extract the volatiles and then identify them by separation with chromatographic techniques [5]. Unfortunately, this takes a significant amount of time, trained personnel and expensive equipment that is only accessible in a laboratory. This type of analysis can therefore be done only on a sample basis and guarantees the freshness of the products only in a statistical way. Monitoring the agri-food chain in a widespread manner requires the creation of sensors that are small, cheap and fast.

Gas sensors are less invasive than other types of sensors and resistive devices are usually simpler and cheaper. Metal oxide chemoresistors are ideal candidates for this purpose: their size is a few microns and they are cheap because they are very simple. After thick and then thin films [16], the latest generation uses nanostructures, i.e., structures in which at least one dimension is of the order of nanometers [17]. The most commonly used nanostructures are nanowires (quasi-one-dimensional structures). The tiny diameter of the nanowires (NWs) causes the interaction on their surface to affect a large part of the wire section [18]. This way, the response is much higher and the limit of detection (LoD) is in parts per billion (ppb). Silicon and metal oxide nanowires have been studied as ammonia sensors [19,20,21,22,23,24]. Nanowires are commonly used as a porous thin film on which metal electrodes are deposited [25,26] but they can also be grown directly from the electrodes [27,28] or even contacted individually [29]. A single nanowire has already been used to measure the freshness of mackerel samples but used in a traditional way as a simple chemiresistor [30].

In this work, a single tin oxide nanowire was used and made to work at three different temperatures. The responses were combined in a virtual array, which, working as an electronic nose, was able to evaluate the freshness of the tested fish. The response of the gas detection system to the TVB-N was compared with the total life count, proving capable of measuring the freshness of the rainbow trout quickly and precisely.

## 2. Materials and Methods

### 2.1. Synthesis of SnO_2_ Nanowires

A forest of tin oxide (SnO_2_) nanowires was grown by a chemical vapor deposition. An alumina boat filled with pure tin monoxide was used as the evaporation source, placed in the center of a horizontal quartz tube inside a Lindberg Blue M oven (Thermo Fisher Scientific, Waltham, MA, USA) at its maximum temperature. A piece of silicon wafer (about 1 × 3 cm^2^) was deposited with a thin gold film (about 5 nm) and placed 1 cm from the alumina boat. Silicon and gold, respectively, act as a substrate and catalyst for the growth of nanowires. The quartz tube was pumped to 10^−2^ mbar and purged with high-purity argon (99.999%) three times and then the system was pumped up to its pressure limit. The temperature was raised from room temperature to 850 °C with a ramp of 25 °C per minute and the oven was left at 850 °C for five minutes. A flow of 0.35 standard cubic centimeters of oxygen then flowed through the system, starting the process. The growth process lasted 30 min and finally the system was shut down and allowed to cool. At the end of the process, the samples were covered with a soft and homogeneous white layer composed of SnO_2_ nanowires.

### 2.2. Material Characterization

The CVD-grown tin oxide nanowire forest was characterized by X-ray diffraction (XRD) using a Philips Xpert Pro (Malvern Panalytical, Malvern, UK) operating at 40 kV with CuKα radiation. Secondary electron microscopy (SEM) and transmission electron microscopy (TEM) images were acquired with a Hitachi S-4800 (Tokyo, Japan) and a JEM-100CX (JEOL, Tokyo, Japan), respectively.

### 2.3. Fabrication of the Sensor

A square of the substrate with the forest of nanowires (approximately 1 × 1 cm^2^) was sonicated in dimethylformamide for two seconds and the resulting dispersion was drop cast onto a Si/SiO_2_ wafer by spinning it at 6000 rpm. An array of Ti/Pt (10/250 nm) electrodes was deposited on top of the dispersed nanowires using UV lithography. Using a resistance measurement and optical microscopy, pairs of adjacent electrodes connected by nanowires were found. These electrode pairs were characterized by SEM to find cases where a single nanowire was connecting the metal pads.

### 2.4. Gas Sensor Measurements

The single nanowire sensor was tested in a system consisting of a measuring chamber with a heatable holder and microprobes. The measuring chamber was connected to high-purity gas cylinders through mass flow controllers. The microprobes were connected to a multimeter (Keithely 2410, Cleveland, OH, USA) interfaced with a data acquisition program (LabView, National Instruments, Austin, TX, USA). Initially the device was kept at 500 °C in nitrogen for 4 h while it was powered at 1 V in order to stabilize the nanostructures and their intrinsic resistance. This procedure served to stabilize the electrical properties of the nanostructures so that they did not change over time [17]. The electrical contact of the semiconductor nanowires with the metal electrodes was studied by analyzing the I-V curves. The good linear behavior found proved a good ohmic contact.

The sensor worked under a voltage of 1 V at three different temperature values (200, 250, 300 °C) towards low concentrations of ammonia (0.1–5 parts per million, ppm) with a total gas flow maintained at 400 sccm. The sensor response was calculated with the standard definition S = R_air_/R_gas_, where R_gas_ and R_air_ were, respectively, the resistance of the sensor in the presence of ammonia and in air. The response time of the device was also calculated in the standard way as the time it took to reach 90% of the maximum response. Similarly, the recovery time was calculated as the time to reach 90% of complete recovery. The limit of detection (LoD) was calculated as 3·SD_noise_/sensitivity, where SD_noise_ was the standard deviation of the sensor signal and sensitivity was the derivative of the sensor response as a function of the gas concentration [31].

### 2.5. Trout Spoilage Measurements

The fresh rainbow trout fish were purchased from a fish farm in Verona (Italy) and kept on ice for less than 1 h upon arrival in the laboratory. Several pieces of trout weighing 20 g were cut from the fresh fish using disposable gloves and autoclaved tools. Each piece was stored in a separate vessel until the measurement with the gas sensor. A few samples were stored at room temperature (25 °C) and a few in the refrigerator (4 °C). A sample was placed in the sensing chamber initially every hour then every three hours and finally every six hours to measure the emitted TVB-N. Immediately after measuring with the gas sensor, the sample was subjected to a microbial analysis in order to compare the two measurements. The total viable count (TVC) was performed using a spread plate technique [16] on a plate count agar and agar base (Oxoid CM0463 and 0055, Hampshire, UK). The plates were counted after an incubation time of 48 h at 30 °C.

### 2.6. Multivariate Statistics and Data Mining

A principal component analysis (PCA) was applied to the response values of the gas sensor at three different temperatures combined together. In this case, the PCA did not reduce the dimensionality but only served to visualize the spatial relationships between the points in a more evident way. The same three-dimensional points were used to quantitatively estimate the TVC value of the fish samples by means of a linear kernel support vector machine [32] used as a regressor. The points measured (double-blind) were randomly divided into two sets, train (32 points) and test (18 points), in order to calibrate the system and then evaluate its quantification performance.

## 3. Results and Discussion

### 3.1. Characterization of the Nanowires

**Figure 1:**
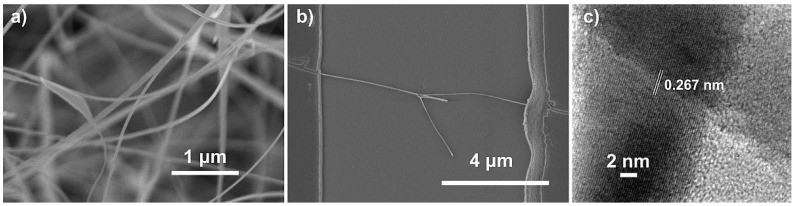
(**a**) SEM image of the SnO_2_ nanowires grown by CVD (magnification 30 k); (**b**) SEM image of the sensor: a single SnO_2_ nanowire bridging the metallic electrodes on the sides (magnification 12 k); (**c**) TEM image of two crossing nanowires.

The morphology of the spaghetti-like SnO_2_ nanowires obtained by CVD was studied by scanning electron microscopy. An SEM image of the nanowire layer is shown in Figure 1a.

Figure 1a shows long and thin nanowires with a constant diameter whose average value was 40–80 nm. The SEM image in Figure 1b shows the single nanowire that was used as a sensor by connecting the two electrodes to the sides. The nanowire forks in the center of the space between the two electrodes. The diameter of the nanowire was approximately 57 nm on the left side and 33 nm on the right side. The thin diameter of the right side and the probable potential barrier in the center contributed to the improvement of the sensor performance. Figure 1c shows a TEM image of two crossing nanowires. The interplanar fringes of 0.267 nm corresponded with the crystalline planes (101) of the tetragonal SnO_2_ structure. The image confirms that the nanowires were monocrystalline with no amorphous layers.

**Figure 2:**
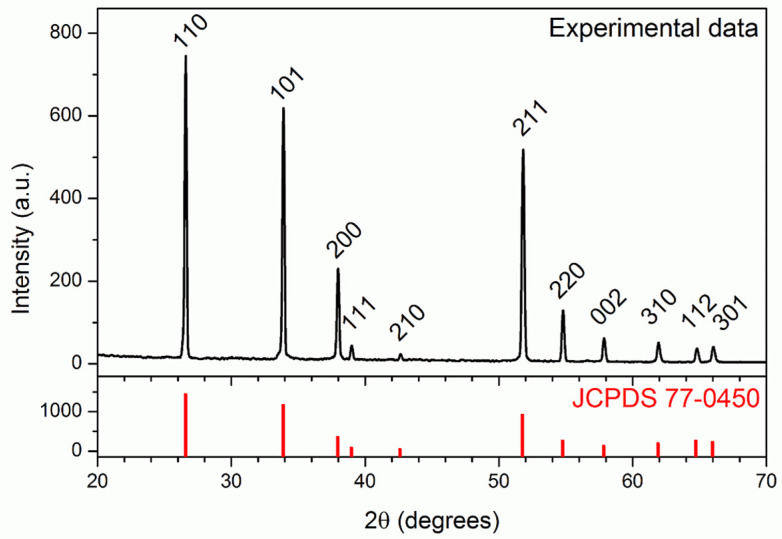
XRD pattern of SnO_2_ nanowires grown on the substrate (one of which was used as a single nanowire sensor). The tetragonal SnO_2_ reference pattern (JCPDS 77-0450) is shown below (red in line).

The composition and structure of the SnO_2_ nanowires were also confirmed by the X-ray diffraction pattern shown in Figure 2.

All of the diffraction peaks present in the pattern could be easily indexed to the tetragonal phase of SnO_2_ with lattice parameters of a = b = 4742 Å and c = 3186 Å and therefore agree well with the standard values (JCPDS n. 77-0450). The absence of amorphous contributions, impurity peaks or other SnO_2_ phases confirmed the high purity of the nanowires.

### 3.2. Ammonia-Sensing Performance

**Figure 3:**
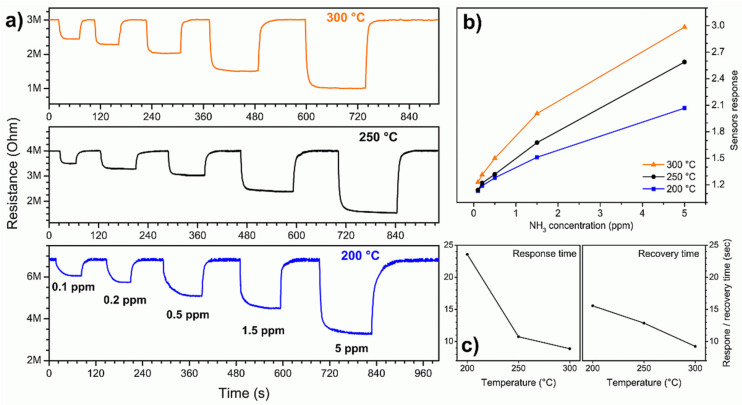
(**a**) Dynamic resistance at three temperature values during the injection of different concentrations of ammonia; (**b**) sensor response as a function of the ammonia concentration for different working temperatures; (**c**) response and recovery times as a function of the sensor working temperature.

The sensor performance was initially tested with low ammonia concentrations (0.1 to 5 ppm). The dynamic resistance of the sensor was tested at three different temperatures (200, 250 and 300 °C). The three answers obtained then composed the 3D signal processed by the machine learning algorithms. The dynamic resistance plots at different temperatures are shown in Figure 3a.

The resistance of the nanosensor was constant in air and, at any working temperature, it dropped sharply when ammonia gas was flushed into the chamber. When the ammonia flow was stopped and pure air was returned into the system, the resistance returned to its original value. This behavior is typical of *n*-type semiconductors [17], in response to reducing gases such as ammonia [18]. The detection mechanism is known in the literature: when the nanowire is exposed to air, oxygen is adsorbed in the form of O^−^ and O^2−^, draining electrons from its interior to form chemical bonds on the surface. Decreasing the number of charge carriers increases the sensor resistance. When the ammonia molecules landed on the surface of the nanostructure, they reacted with the adsorbed oxygen atoms breaking their chemical bond and releasing electrons into the nanowire. The increase in the number of charge carriers decreased the resistance of the sensor. The three graphs in Figure 3a show that the resistance variation was proportional to the ammonia concentration. It can also be seen how, as the temperature increased, the air resistance of the sensor decreased and the response and recovery became faster. Figure 3b shows the sensor response (calculated as explained in Section 2.4) as a function of the gas concentration for the three temperatures tested. The response increased with the concentration almost linearly and was greater for higher working temperatures. The speed of the sensor is quantified in Figure 3c where the average response time and recovery time at each temperature are shown as defined in Section 2.4. Both times decreased according to the working temperature. The response time was higher than the recovery time at 200 °C but at higher temperatures it became shorter or comparable. In general, the response and recovery times were very fast: at the lowest temperature they were, respectively, 24 and 15.5 s while at higher temperatures they were always less than 13 s. The limit of detection (calculated as specified in Section 2.4) was very low at all temperatures tested: 13.4, 4.9 and 1.8 ppb at 200, 250 and 300 °C, respectively [31].

### 3.3. Trout Fish Spoilage Measurements

**Figure 4:**
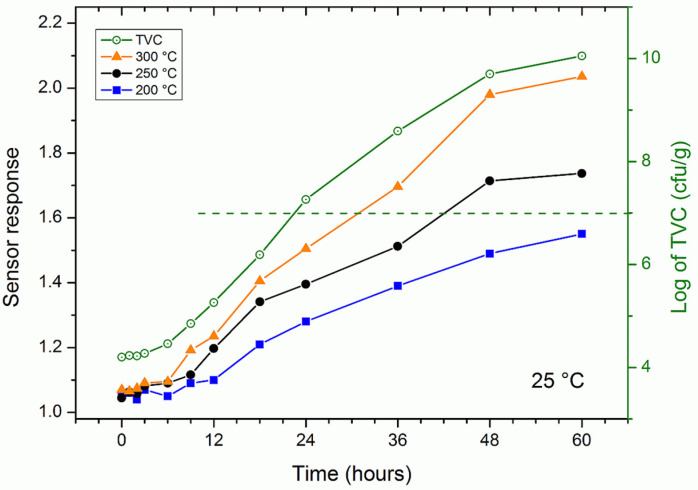
Sensor response (solid symbols, left scale) and bacterial population (green open circles, right scale) in fresh trout fish kept at room temperature (25 °C) over a period of 60 h.

The sensor was then used to measure the freshness of the rainbow trout samples stored at 25 °C. As the sensor measured the volatiles emitted by the fish sample (mainly TVB-N, i.e., ammonia, dimethylamine and trimethylamine), it was not possible to compare the response with a known concentration. For this reason, together with the sensor response, Figure 4 also shows the microbial count used as a reference.

The responses of the gas sensor are read on the left scale while the total viable count is on the right of Figure 4. The response of the sensor increased as the working temperature increased, as in the case of ammonia. At all temperatures, the response increased over time, slowly over the first six hours and then faster. The TVC increased similarly, starting at a value of 4.2 (note that the log of the TVC was plotted), reaching the maximum slope at around 20 h and exceeding a value of 10 after 60 h. The response of the single nanowire resistive sensor could be considered to be a good indirect measure of the microbial count and therefore of the freshness of the fish. The dashed horizontal green line identifies the threshold considered as the end of the shelf life of the fish both in literature [33,34] and for the authorities [35,36]. The threshold was exceeded approximately after 22 h and 40 min of storage at room temperature (25 °C).

**Figure 5:**
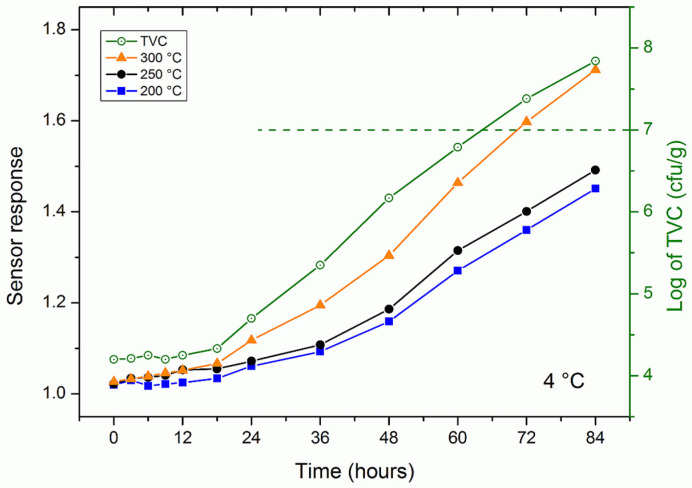
Sensor response (solid symbols, left scale) and bacterial population (green open circles, right scale) in fresh trout fish kept at 4 °C over a period of 60 h.

Also in this case the responses of the gas sensor are read on the left scale, while the total viable count on the right. The sensor was also tested with rainbow trout samples stored at 4 °C for 84 h. Figure 5 shows the sensor response at 200, 250 and 300 °C and the microbial count detected on the samples over time. During the first 12 h, the microbial count remained more or less constant at around a value of 4.2, then began to rise almost linearly to reach a value of 7.84 after 84 h. The edibility threshold in this case was reached after approximately 64 h. The response of the gas sensor behaved in a similar way at all temperatures.

The trend of the response of the gas sensor at the various temperatures in Figure 4; Figure 5 was very similar to that of the microbial count. This could be explained by the fact that TVB-N is the metabolic product of the microbes responsible for the degradation of fish [37] and meat [38].

**Figure 6:**
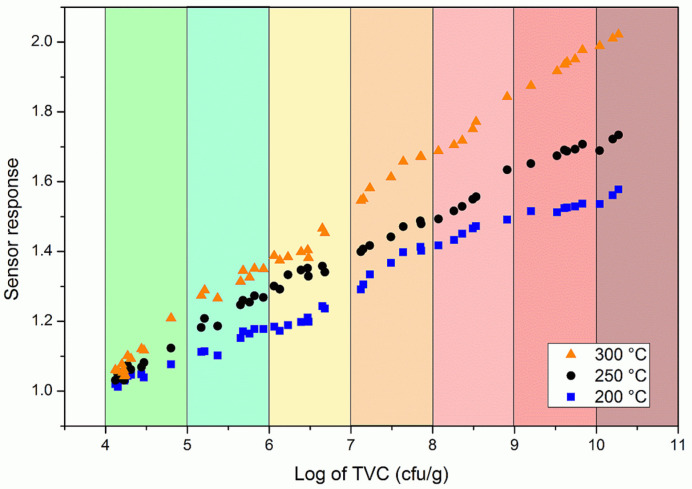
Double-blind measurements of the sensor response as a function of the total viable count in rainbow trout samples.

To evaluate how effective the response of the single nanowire gas sensor could be as a measure of fish freshness, a series of samples stored at different temperatures were measured in a double-blind capacity. For each sample, the response of the gas sensor was first measured and immediately afterwards the microbial analysis was carried out in order to have a comparison under the same conditions. The measurements are shown in Figure 6 where the response is reported as a function of the logarithm of the total viable count. The response was linear at all working temperatures with Pearson’s correlation coefficients of r were greater than 0.99 in all cases. The error decreased as the temperature rose but was always less than 10%. This demonstrated that the sensor response could be considered to be a good indirect measure of the TVC.

The three responses of the single nanowire gas sensor were combined to obtain a sort of virtual electronic nose following the approach already tested previously [39]. The 3D points obtained also included the correlations between the various answers and were therefore much more informative than a single response. A principal component analysis (PCA) is commonly used to increase the interpretability while minimizing information loss [40]. In this case, the three-dimensional points were processed with the PCA in order to visually evaluate how they were spatially correlated.

**Figure 7:**
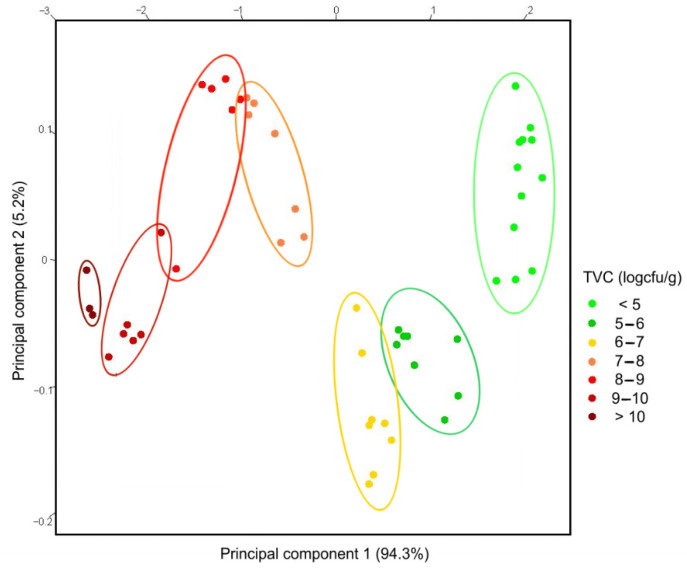
PCA plot of random samples of rainbow trout. The color indicates the log(TVC) with the same scale of the X-axis in Figure 6.

The PCA graph in Figure 7 shows the points divided by color according to the TVC value. Each group of points is colored according to a unit interval of the logarithm of the TVC, as shown in Figure 6. It can be seen that the points are arranged in a zigzag line that goes down to a log value (TVC) of 6–7, then goes up to 8, goes down to 10 and then goes up again. Each group of points is well separated from the others, with a possible small overlap only with the immediately preceding or following group.

This overlap was expected as the measurement of the microbial count was continuous and therefore the points along the zigzag line ought to have also been continuous. This was evident in the point clouds of the intervals 7–8, 8–9 and 9–10, which concatenated well along the imaginary zigzag line.

Figure 7 demonstrates that the single nanowire sensor was very sensitive and accurate as it not only distinguished the spoiled fish samples (over the threshold of 10^7^ cfu/g) but also the various stages of the degradation process as measured by the total viable count.

**Figure 8:**
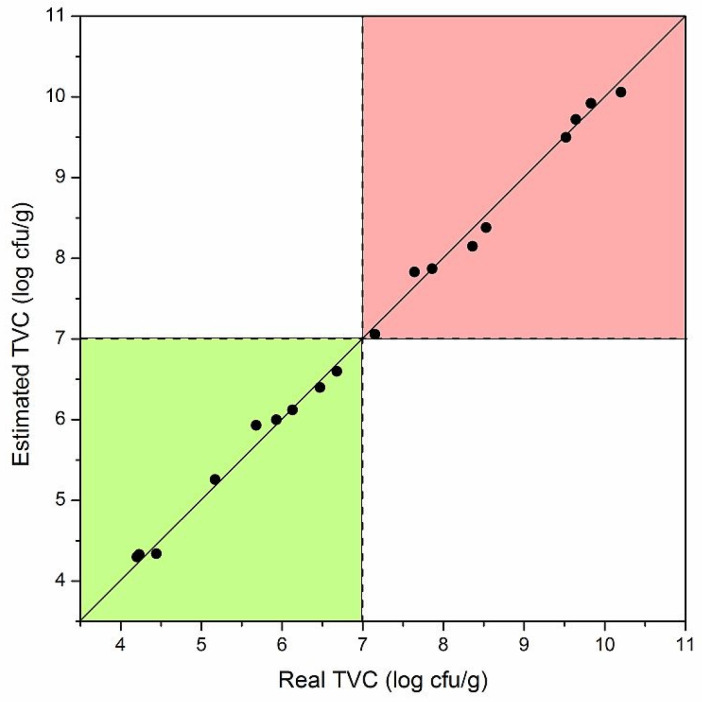
Estimates of the TVC values versus the actual measured TVC values for random rainbow trout samples. The green color indicates the area in which the microbial load was compatible with consumption while the red one indicates that the fish had deteriorated.

To obtain an automatic quantitative estimate by the nanosensor, a support vector machine was used as a regressor [29]. In this way, the three responses of the gas sensor were automatically transformed into an estimate of the TVC and therefore of the degree of freshness of the fish. Figure 8 shows the regressor estimates against the “true” values (obtained from the TVC measurements). Clearly, the diagonal represents an estimate identical to the TVC value and therefore a perfect functioning of the nanosensor.

The points in Figure 8 are all very close to the diagonal, indicating a very good estimate of the freshness of the fish. The average error obtained on all points was less than 5%, demonstrating that the single nanowire gas sensor approximated the microbial count measurement very well.

The points were collected in the two colored areas. The green zone indicates a TVC value that allowed the consumption of fish while the red zone indicates that the fish had deteriorated. This again meant that the sensor was in perfect agreement with the microbial count and could be used as a tool to ascertain the freshness of the rainbow trout samples. It should be noted that the results were obtained under laboratory conditions with samples collected from only one fish. It is reasonable to expect a larger error when working in the field on different fish.

The single nanowire gas sensor is in fact tiny and portable (while the TVC can only be done in a laboratory equipped by trained personnel) and takes less than half a minute (while the TVC usually takes days). For this reason, we consider that the proposed sensor could be ideal to assess the freshness of fish during its shelf life.

## 4. Conclusions

A SnO_2_ single nanowire gas sensor was used to assess the deterioration of rainbow trout fish. The performance was initially tested by measuring ammonia concentrations from 0.1 to 5 ppm at three different operating temperatures. The sensor responded quickly even at very low concentrations. The responses of the nanosensor at different temperatures were then used to monitor trout spoilage over time. The gas sensor response proved to be a good approximation of the total viable count at all working temperatures. The use of machine learning algorithms allowed the determination of the spoilage stage of the fish and the estimation of its total viable count. Considering the tiny size (tenths of a millimeter), economy, ease of use and speed, the single nanowire gas sensor would be ideal as a non-invasive tool for monitoring the freshness of rainbow trout along its production and distribution chain.

## Data Availability

The data presented in this study are openly available in OSF with doi:10.17605/OSF.IO/F6CSX.
